# Deep Eutectic Solvents for Biodiesel Purification in a Microextractor: Solvent Preparation, Selection and Process Optimization

**DOI:** 10.3390/bioengineering9110665

**Published:** 2022-11-08

**Authors:** Sara Anđelović, Marko Božinović, Željka Ćurić, Anita Šalić, Ana Jurinjak Tušek, Kristina Zagajski Kučan, Marko Rogošić, Mia Radović, Marina Cvjetko Bubalo, Bruno Zelić

**Affiliations:** 1 Faculty of Chemical Engineering and Technology, University of Zagreb, Marulićev trg 19, HR-10000 Zagreb, Croatia; 2 Faculty of Food Technology and Biotechnology, University of Zagreb, Pierottijeva ul. 6, HR-10000 Zagreb, Croatia; 3 Department of Packaging, Recycling and Environmental Protection, University North, Trg dr. Žarka Dolinara 1, HR-48000 Koprivnica, Croatia

**Keywords:** biodiesel, DES, microextractor, purification, glycerol extraction

## Abstract

The most important and commonly used process for biodiesel synthesis is transesterification. The main by-product of biodiesel synthesis by transesterification is glycerol, which must be removed from the final product. Recently, deep eutectic solvent (DES) assisted extraction has been shown to be an effective and sustainable method for biodiesel purification. In this study, biodiesel was produced by lipase-catalysed transesterification from sunflower oil and methanol. A total of 12 different eutectic solvents were prepared and their physical properties were determined. Mathematical models were used to define which physical and chemical properties of DES and to what extent affect the efficiency of extraction of glycerol from the biodiesel. After initial screening, cholinium-based DES with ethylene glycol as hydrogen bond donor was selected and used for optimization of extraction process conditions performed in a microsystem. To determine the optimal process conditions (temperature, biodiesel:DES volume ratio, residence time), the experimental three-level-three-factor Box-Behnken experimental design was used. In the end, a combination of a mathematical model and experimental results was used to estimate how many micro-extractors are necessary for the complete removal of glycerol.

## 1. Introduction

Biodiesel, as one of the representatives of biofuels, still has one of the largest shares of renewable energy sources. The most common method of producing biodiesel is transesterification. When an enzyme is used as a catalyst, the production can be said to be green and environmentally friendly. Since biodiesel produced by transesterification is not suitable for direct use in internal combustion engines, as it may contain traces of soap, catalyst, methanol (and other alcohols), metals, water, oil, and glycerides, it must be purified to meet the appropriate quality standards (ASTM D6751, EN 14214) [1,2]. Moreover, the downstream process of biodiesel production largely determines the final price of biodiesel [3]. This is due to the industrial purification method for biodiesel, wet washing, which has the major drawback of consuming about 10 L of water per 1 L of purified biodiesel [2]. In addition to the fact that wet washing of biodiesel consumes a large amount of fresh water, the wastewater after biodiesel purification must be cleaned before it is discharged into the environment, which requires a large amount of energy. Therefore, it is necessary to find more environmentally friendly methods for the purification of biodiesel and to make the whole production/purification process green.

Because of all the disadvantages of the wet washing, various other methods have been developed, such as dry washing, membrane separation, liquid-liquid extraction, precipitation, reactive distillation, and complexation [4,5,6,7]. Liquid-liquid extraction with deep eutectic solvents (DESs) is one of the alternative methods to remove glycerol from biodiesel after the transesterification [8]. DESs have unique properties that include ease of preparation, low cost, environmental friendliness compared to conventional solvents, non-flammability, low volatility, high dissolving power, and good biodegradability. Biodegradability and environmental impact are largely related to the character of the individual pure components that make up DESs [9]. Another important property of DESs is their chemical adaptability, which means that eutectic solvents can be developed for specific applications [10].

Shahbaz et al. [11] synthesized various DESs and removed all the free glycerol from palm oil-based biodiesel with an optimal molar ratio of DES to biodiesel of 1:1. In the work presented by Naiwanti and Zullaikah [12], the authors used cholinium-based DES with ethylene glycol as hydrogen bond donor to remove the total glycerol, but the amount of total glycerol still remained above the specification standard. The same effect was found in the work of Petračić et al. [13] where the same DES (molar ratio 1:2.5) was used as an extraction medium in batch experiments and in the continuous Karr column. It was found that after extraction, the free glycerol content was below the limit for all samples, but the total glycerol and glycerides content was too high to fully meet biodiesel quality standards.

In addition to using DES instead of water to purify biodiesel, switching from batch to continuous processes could also improve the purification efficiency. Microextractors, miniaturized systems, are an example of how smaller is better. Due to their internal properties—large surface-to-volume ratio, intensification of mass transfer, and small dimensions—the interphase diffusion can be neglected [14,15]. In the work of Šalić et al. [8] the authors tested an extraction efficiency of seven different DESs based on mixtures of choline chloride with ethylene glycol or glycerol for glycerol extraction from raw biodiesel produced by transesterification using three microextractors of different sizes. With a residence time of only 13.61 s, glycerol was almost completely removed from biodiesel using a cholinium-based DES with glycerol as a hydrogen bond donor. The results obtained clearly indicate that the microextractors can be used for glycerol removal from raw biodiesel.

As can be seen from the literature, the most commonly used solvents for the removal of glycerol from biodiesel were DESs based on choline chloride and ethylene glycol or glycerol as hydrogen bond donors in various molar ratios. However, DESs can also be formed by mixing other components [9]. The chemical structure of hydrogen donor and acceptor has a significant effect on the formation, properties and stability of the deep eutectic solvent [9]. In the preparation of eutectic solvents, it is necessary to know certain factors such as the purity of the components and the water content of each component, as well as the storage and drying of the prepared DESs, so that their physicochemical properties do not change. Small changes in the water content of DES can lead to significant differences in physicochemical properties such as viscosity, density or polarity. Water can disrupt the hydrogen bond acceptor/donor network because it can act as both a hydrogen bond acceptor and donor. For this reason, and to ensure consistency of properties, different approaches are used for the preparation of DESs [16,17,18,19,20].

In this work, raw biodiesel was produced by transesterification. Glycerol was then removed from the product using 12 different DESs in microextractors, and the efficiency of the extraction was assessed. For the DES exhibiting the highest extraction efficiency, extraction process was optimized using the Box-Behnken model at three levels and with three factors. After process optimization, extraction was performed in a microextractor under optimal process conditions. Finally, a mathematical model was used in order to estimate how many microextractors are required for the complete removal of glycerol from biodiesel.

## 2. Materials and Methods

### 2.1. Materials

#### Chemicals

Edible sunflower oil (Zvijezda, Zagreb, Croatia) was purchased at a local supermarket. Commercial lipase from *Thermomyces lanuginosus* (Lipolase 100L), ethylene glycol, glycerol, betaine and propylene glycol were purchased from Sigma-Aldrich Handels GmbH (Saint Louis, MO, USA). Methanol was purchased from BDH Prolabo (Lutterworth, United Kingdom). Choline chloride, zinc chloride and propylene glycol were purchased from Acros Organics (Geel, Belgium). Ethanol was purchased from Gram-mol (Zagreb, Croatia). The chemicals were of analytical grade and were used without further purification, except for drying.

### 2.2. Methods

#### 2.2.1. Production of Biodiesel in a Batch Reactor

Biodiesel production from edible sunflower oil using commercial free lipase was performed in a glass jacketed batch reactor (*V* = 500 mL). The total mass of the mixture was 275.48 g, and the mixture consisted of 225 g sunflower oil, 27.98 g methanol, and 22.5 g lipase enzyme solution [21]. The enzyme solution was prepared by mixing commercial lipase with 0.01 mol/L phosphate buffer pH 7.4 at a volume ratio of 1:10. The transesterification reaction was started by adding the enzyme to the reaction mixture and carried out at a temperature of 40 °C (optimal temperature for the lipase-catalyzed transesterification reaction) which has kept constant by a water bath (Thermomix 1420, Braun, Germany). The process lasted 48 h. Mixing was carried out by a magnetic stirrer (MS-H-S, DLAB, Rowland St, City of Industry, CA, USA) at 600 rpm. At the end of the reaction, the mixture was transferred to a separating funnel to separate glycerol at the bottom. In the further course of the research, the upper phase was used, i.e., partially purified biodiesel [8].

#### 2.2.2. Preparation of Deep Eutectic Solvents

Choline chloride, glycerol, ethylene-glycol, betaine, propylene-glycol and zinc chloride were used in waterless DESs preparation. Prior to DESs preparation, the DESs components were dried in a vacuum oven at 60 °C for 8 h. DESs were prepared by mixing dried chemicals in different molar ratios (Table 1) in a glass vial equipped with a stopper. The components were mixed on a magnetic laboratory stirrer (Rotamix S-10, Tehtnica, Železniki, Slovenia) at 700 rpm and 70–90 °C until a homogeneous, colorless liquid was obtained [22,23,24,25].

#### 2.2.3. Measurements/Determination of Physico-Chemical Properties of Prepared Deep Eutectic Solvents

The physico-chemical properties of the prepared DESs were determined at 25 °C. Specific conductivity was determined with a conductivity meter (Schott Instruments Lab 960, Mainz, Germany, resolution 0.01 for a range of 0.00–19.99 mS cm^−^^1^, 0.001 for a range of 0.000–1.999 μS cm^−^^1^), refractive index was measured with a refractometer (Abbe RL-3, Kern, Myszków, Poland, accuracy ±0.0002), and a density meter (Anton Paar DMA 4500 M, Graz, Austria) was used for density measurements. A programmable rheometer (Brookfield DV-III Ultra, East Lyme, CT, USA, accuracy ±1%) was used to determine dynamic viscosity. As for thermal properties, thermal conductivity, thermal diffusivity and heat capacity were measured with a thermal conductivity meter (Linseis Transient Hot Bridge 1, Selb, Germany, measurement uncertainties according to ISO standards) [22,23,24,25].

#### 2.2.4. Calculation of Deep Eutectic Solvents Descriptors

DESs chosen for glycerol extraction were mathematically described using *σ*-profiles defined with the COSMOtherm software and the generated DES descriptors were further used to mathematically model the glycerol extraction efficiency.

DESs constituents were initially optimized both from an energy and geometry point of view in TURBOMOLE software by adopting DFT (density functional theory) with BP86 functional level of theory and def-TZVP basis set. Molecules consisting of two or more ions (e.g., choline chloride) were treated as ion pairs and their structures were optimized according to Abranches et al. [26]. These quantum chemical calculations resulted in the software-generated .cosmo file for each optimized molecule that was further used in BIOVIA COSMOtherm software. The files contained *σ*-profile curves that provided a quantitative representation of the molecules’ polar surface screen charge on the polarity scale, and therefore included all information necessary for the calculation of the σ-profile function and σ-profile descriptors. As the calculation output, *σ*-profile for each molecule was created. The *σ*-profile curve for each molecule was divided into ten regions, making the region width of 0.005 e / Å^2^ and covering the total range from –0.025 to +0.025 e/Å^2^. The areas under the curve were integrated separately for each defined region. This was done by simple summation of tabulated *σ*-profile data point ordinate values as presented by the COSMOtherm software. The ordinate values lying on the boundaries of the regions were split into halves, and each half was attributed to one of the neighboring regions. Thus, 10 *S*-descriptors (*S*^1^–*S*^10^) of *σ*-profiles were calculated to represent these 10 areas numerically.

For the preparation of the DESs descriptor set, the DESs were modelled as a molar mixture of hydrogen bond acceptor and donor according to Table 1. Any change in DES composition can be described by a change in its *σ*-profile and the associated numerical value of its descriptors. To obtain a unique descriptor set for each particular DES, the *σ*-profiles of its constituents were processed as follows. The descriptors of the studied DES (*S^i^*_mix_) were calculated from the hydrogen bond acceptor and donor descriptors according to Equation (1) proposed by Benguerba et al. [27]:

(1) $$S_{\mathrm{mix}}^{i}=\sum_{j=1}^{NC}X_{j}S_{\sigma -\mathrm{profile},j}^{i}$$

where *i* denotes the descriptor number (1–10), *j* stands for the DES constituent number, *X_j_* is the molar fraction of each constituent, *S^i^_σ_*_-profile,*j*_, is the *i*-th descriptor of *j*-th constituent, and *NC* is the total number of constituents from which DES is prepared.

#### 2.2.5. Two-Phase Liquid-Liquid Extraction in a Microextractor

**Figure 1:**
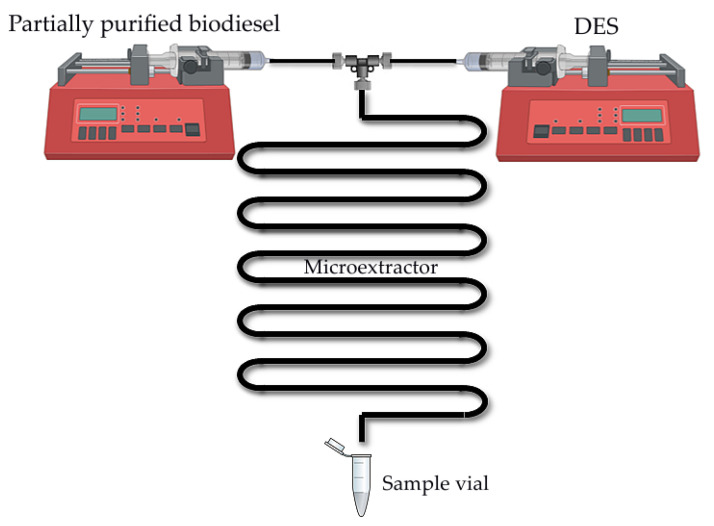
Scheme of the experimental set-up used for the biodiesel purification process in a microextractor.

The extractions (Figure 1) were performed in a PTFE (polytetrafluoroethylene) microextractor with two inlets and a T-junction (*L* = 30 cm, *d* = 1000 µm). Liquids were fed to the microextractor using syringes mounted on two piston pumps (Harvard PHD 4400 Programmable, Harvard Apparatus, Inc, Holliston, MA, USA) connected with PTFE tubing. One syringe was filled with crude biodiesel, and the second syringe contained DES. The flows of biodiesel and DES were adjusted to achieve the 1:1 volume ratios of components in a microextractor. Extractions were performed for different residence times (0.05–30 min) depending on the experiment. Samples were collected at the exit of the microextractor in Eppendorf test tubes. The samples collected in this way were centrifuged (Universal 320 R, Hettich, Buford, GA, USA; 14,000 rpm, *T* = 25 °C, *t* = 15 min) to completely separate the biodiesel (upper layer) from the DES (lower layer). The samples were collected from upper phases using a needle to avoid phase contamination. Samples were diluted with ethanol and the influence of solvents on glycerol extraction was monitored according to the method described in Section 2.2.6.

From the values obtained, biodiesel yield (*Y*), glycerol extraction efficiency (*E*), distribution coefficient (*K*_P_) and mass fraction of glycerol in biodiesel (*w*) were calculated according to the following equations (Equations (2)–(5)):

(2) $$Y=\frac{\gamma_{B,\;P.B.}}{\gamma_{B,\;C.B.}}$$

(3) $$E=1-\frac{\gamma_{G,P.B.}}{\gamma_{G,C.B.}}$$

(4) $$K=\frac{\gamma_{G,C.B.}-\gamma_{G,P.B.}}{\gamma_{G,C.B.}}$$

(5) $$w=\frac{\gamma_{G,P.B.}\cdot V_{B}}{m_{B}}\;$$

where G denotes glycerol, B denotes biodiesel, P.B. denotes purified biodiesel, *γ* denotes mass concentration (mg/mL), *V* denotes volume (mL) and C.B. denotes crude biodiesel.

#### 2.2.6. Determination of Glycerol and FAME Concentration in the Samples

The concentrations of free fatty acids esters (FAME) and glycerol in the samples before and after extraction were determined using a GC (Shimadzu GC-2014, Kyoto, Japan) gas chromatograph equipped with FID detector and Zebron ZB-wax GC capillary column (length 30 m, i.d. 0.53 mm and film thickness 1.00 μm, Phenomenex, Aschaffenburg, Germany) by the method described elsewhere [28]. To confirm repeatability, every sample was analyzed in triplicate. On a 95% confidence interval, the results showed no significant difference.

#### 2.2.7. Data Analysis and Mathematical Modeling

##### Modeling of Extraction Efficiency Based on DESs Descriptors and Physical Properties

Glycerol extraction efficiency (*E*) was modeled as the function of *σ* profile of the DES expressed by a set of *S^i^*_mix_ descriptors according to Equation (6):

(6) $$E=f\left(S_{\mathrm{mix}}^{i}\right)$$

*S^i^*_mix_ descriptors for the description of glycerol extraction efficiency were selected based on the Person correlation matrix. Based on significant correlations, *S*^1^_mix_, *S*^3^_mix_, *S*^4^_mix_, *S*^5^_mix_ and *S*^6^_mix_ were used as the models input variables. The relationship between glycerol extraction efficiency and selected *S^i^*_mix_ descriptors was modeled using multiple linear regression (MLR) according to Equation (7), nonlinear regression (NLR) according to Equation (8) and piecewise linear regression (PLR) according to Equation (9):

(7) $$E=b_{0}+b_{1}\cdot S_{\mathrm{mix}}^{1}+b_{2}\cdot S_{\mathrm{mix}}^{3}+b_{3}\cdot S_{\mathrm{mix}}^{4}+b_{4}\cdot S_{\mathrm{mix}}^{5}+b_{5}\cdot S_{\mathrm{mix}}^{6}$$

(8) $$E=b_{0}\cdot {\left(S_{mix}^{1}\right)}^{b_{1}}\cdot {\left(S_{mix}^{3}\right)}^{b_{2}}\cdot {\left(S_{mix}^{4}\right)}^{b_{3}}\cdot {\left(S_{mix}^{5}\right)}^{b_{4}}\cdot {\left(S_{mix}^{6}\right)}^{b_{5}}$$

(9) $$E=\left\{\begin{array}{ccc} \left(b_{01}+b_{11}\cdot S_{\mathrm{mix}}^{1}+b_{21}\cdot S_{\mathrm{mix}}^{3}+b_{31}\cdot S_{\mathrm{mix}}^{4}+b_{41}\cdot S_{\mathrm{mix}}^{5}+b_{51}\cdot S_{\mathrm{mix}}^{6}\right) & \forall & \left(E\leq b_{n}\right)\\ \left(b_{02}+b_{12}\cdot S_{\mathrm{mix}}^{1}+b_{22}\cdot S_{\mathrm{mix}}^{3}+b_{32}\cdot S_{\mathrm{mix}}^{4}+b_{42}\cdot S_{\mathrm{mix}}^{5}+b_{52}\cdot S_{\mathrm{mix}}^{6}\right) & \forall & \left(E>b_{n}\right) \end{array}\right\}$$

Glycerol extraction efficiency (*E*) was also modeled as the function of DESs physical properties: density (*ρ*), dynamic viscosity (*η*), electrical conductivity (*σ*), refractive index (*n*_D_), thermal diffusivity (*a*), thermal conductivity (*λ*), and specific heat capacity (*c_p_*) according to Equation (10):

(10) $$E=f\left(\rho ,\eta ,\sigma ,n_{D},a,\lambda ,c_{p}\right)$$

The relationship between glycerol extraction efficiency and analyzed physical properties of DESs was modeled using multiple linear regression (MLR) according to Equation (11), nonlinear regression (NLR) according to Equation (12), and piecewise linear regression (PLR) according to Equation (13):

(11) $$E=b_{0}+b_{1}\cdot \rho +b_{2}\cdot \eta +b_{3}\cdot \sigma +b_{4}\cdot n_{D}+b_{5}\cdot a+b_{6}\cdot \lambda +b_{7}\cdot c_{P}$$

(12) $$E=b_{0}\cdot \rho^{b_{1}}\cdot \eta^{b_{2}}\cdot \sigma^{b_{3}}\cdot n_{D}{}^{b_{4}}\cdot a^{b_{5}}\cdot \lambda^{b_{6}}\cdot c_{P}{}^{b_{7}}$$

(13) $$E=\left\{\begin{array}{ccc} \left(b_{01}+b_{11}\cdot \rho +b_{21}\cdot \eta +b_{31}\cdot \sigma +b_{41}\cdot n_{D}+b_{51}\cdot a+b_{61}\cdot \lambda +b_{71}\cdot c_{P}\right) & \forall & \left(E\leq b_{n}\right)\\ \left(b_{02}+b_{12}\cdot \rho +b_{22}\cdot \eta +b_{32}\cdot \sigma +b_{42}\cdot n_{D}+b_{52}\cdot a+b_{62}\cdot \lambda +b_{72}\cdot c_{P}\right) & \forall & \left(E>b_{n}\right) \end{array}\right\}$$

The parameters of the MLR models (Equations (7) and (11)), NLR models (Equations (8) and (12)), and PLR models (Equations (9) and (13)) were estimated using the Levenberg-Marquardt algorithm implemented in Statistica 13.0 (Tibco Software Inc, Palo Alto CA, USA). The algorithm searches for numerical solutions in the function parameter space using the least squares method. Calculations were performed in 50 iterations with the convergence parameter of 10^−6^ and 95% confidence interval [29,30].

##### Optimization of Biodiesel Purification in a Microextractor

To optimize the biodiesel purification, the experiments were carried out in the microextractor as described in Section 2.2.5, according to the Box-Behnken experimental design at three levels (−1, 0, 1). The effects of the (i) extraction temperature *X*_1_ (*T* = 25, 40, 55 °C), (ii) residence time *X*_2_ (*τ* = 0.05, 0.5, 0.95 min) and (iii) volume ratio of biodiesel:DES *X*_3_ (9:1, 1:1, 1:9 *v*/*v*) were analyzed. Experimental data were fitted to the second order polynomial equation (Equation (14)):

(14) $$Z=\beta_{0}+\sum_{i=1}^{3}\beta_{i}\cdot X_{i}+\sum_{i=1}^{3}\beta_{ii}\cdot X_{i}^{2}+\sum_{i=1}^{2}\sum_{j=i+1}^{3}\beta_{ij}\cdot X_{i}\cdot X_{j}$$

Therein, *Z* is the predicted response, *β*_0_, *β_i_*, *β_ii_* and *β_ij_* are the regression coefficients for intercept, linear, quadratic and interaction terms, and *X_i_* and *X_j_* are the independent variables. The response surface analysis was performed using Statistica 13.0 (Tibco Software Inc, Palo Alto, CA, USA)

##### Mathematical Modeling of Glycerol Extraction in a Microextractor

The glycerol separation in a microextractor was described with a 2D model including convection in the flow direction (x) and diffusion in two directions (x and y). The mathematical model for steady-state conditions in a microextractor was composed of dimensionless partial differential equations for glycerol concentrations in biodiesel and DES phase and corresponding boundary and initial conditions. The proposed model was described elsewhere [8].

## 3. Results and Discussion

### 3.1. Biodiesel Production in the Batch Reactor

To obtain larger amounts of biodiesel to be used in all purification experiments in this work, biodiesel was synthesized in the batch reactor (*V* = 500 mL) using edible sunflower oil, methanol, and the enzyme lipase from *Thermomyces lanuginosus.* At the end of the reaction, the yield of the obtained biodiesel in the form of FAME was 97.96 ± 2.25% and the glycerol concentration was 117.19 ± 2.70 mg/mL. The obtained results were consistent with those of previous studies [7,21]. Although the yield of FAME in the sample was within the range prescribed by the standards for the quality of biodiesel, the content of glycerol was much higher than allowed (*w* < 0.02%), so biodiesel thus obtained had to be purified. The first and simplest method of glycerol separation was by gravitational settling since glycerol was insoluble in biodiesel. In order to remove most of glycerol, a separation funnel was used. After a settling time of 24 h, samples of biodiesel were taken and the glycerol concentration decreased by 96.96 ± 0.59%, which means that the glycerol concentration in partially purified biodiesel was 3.57 ± 0.78 mg/mL. The same percentage of removed glycerol was obtained in the work of Šalić et al. [8]. The authors reported that several additional steps could be used for biodiesel purification to further increase this percentage, such as second gravitational settling, centrifugation, and filtration, but the total amount of biodiesel removed by these processes would not justify the cost of their application. For this reason, no additional purification steps were performed in this study.

### 3.2. Deep Eutectic Solvents Preparation and Physico-Chemical Properties

As mentioned in the introduction, the most commonly used DESs for the removal of glycerol from biodiesel were DESs based on choline chloride and ethylene glycol or glycerol as hydrogen bond donors in various molar ratios [8,11,12,13]. However, by combining different hydrogen donors and acceptors, DESs with different properties can be obtained. In this research 12 water-free DESs were prepared. They were selected based on their ability to denitrify diesel fuels [22,23,24,25]. Water was not used for DESs preparation to prevent water entering biodiesel because this would require an additional step in the purification, since water must also be removed from biodiesel before use. After preparation, DESs properties: density (*ρ*, g/mL), dynamic viscosity (*η*, Pa s), specific conductance (*σ*, mS/cm), refractive index (*n*_D_, -), thermal diffusivity (*a*, mm^2^/s), thermal conductivity (*λ*, W/(m K)), and specific heat capacity (*c_p_*, J/(g K)) were determined. The selected properties were chosen according to Rogošić and Zagajski Kučan [25], as being among the most important properties relevant to extraction processes. Viscosity is very important when it comes to microfluidics since it is directly connected to the fluid behavior in flow. Also, if the fluids (DESs) are too viscous, they cannot be used in microextractors [31]. As for the density, it determines the settling of the layers of primary and secondary solvent after extraction in macroextractors. Without the differences in the density, two liquids will not separate in the gravitational field. This property is important for macroextractors; however, in a microextractor even the two liquids of the same density flow side by side with a clearly defined boundary when two liquids are introduced into a microchannel by two inlets [32]. The refractive index is important in order to observe the phase separation more easily. With this, and by regulating the flow, it is possible to position the phase boundary between the two liquids at a specific place in the microchannel, which enables complete phase separation at the exit from the microchannel. Knowledge of the electrical conductivity of DES is important for any DES uses that involve electric current, such as the separation by electrocoagulation. Thermal properties are important when extraction is performed at a temperature that is higher or lower than 25 °C [25]. Furthermore, increasing the temperature decreases the viscosity and density of all DESs. In addition, the increase of specific conductance was noted with increasing temperature of the DESs. In the research performed by Abbott et al. [18], it was found that the lower was the viscosity, the higher was the observed specific conductance. Besides heating, a possible strategy to reduce DES viscosity is the simple dilution of DES with water [33,34,35,36] which was not an option in this research.

The obtained results for selected DESs are presented in Table 2.

As can be seen, as the ethylene glycol or glycerol mole fraction increases, the viscosity of the DESs gradually decreases, which is related to the lower viscosity of ethylene glycol and glycerol in comparison to choline chloride. Also, according to literature [37,38,39,40,41,42,43,44], the viscosity of DES is usually higher than 0.1 Pa s which is related to the large network of hydrogen bonds in DESs [45]. Among synthetized DESs, few of them, based on propylene glycol and ethylene glycol, had viscosity below that value which was attributed to the small molecular size of glycol components [46]. As for DESs density, the molar ratio of DES components has a significant effect on their density, which is related to molecular arrangements in DES structure [47].

The comparison of the obtained results with the literature ones was not possible. Namely, the changes in the DES composition (i.e., component ratio, amount of water, purity of components) significantly affect the properties of DES and no literature data were available for DES compositions studied.

### 3.3. Glycerol Extraction in a Microextractor with Different Deep Eutectic Solvents

**Figure 2:**
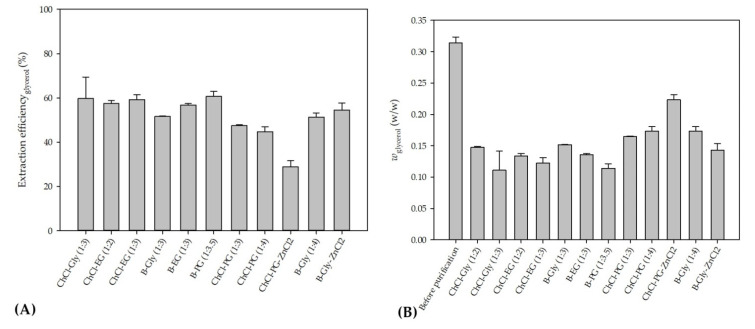
(**A**) Glycerol extraction efficiencies using different DESs and (**B**) mass fraction of glycerol before and after purification for tested DESs.

In order to select the DES which would enable the highest glycerol extraction efficiency, the extraction process was carried out at a temperature of 25 °C, a biodiesel:DES volume ratio of 1:1 and a residence time of *τ* = 0.05 min. The selected volume ratio of biodiesel and DES was based on the literature data [11] where all free glycerol from palm oil-based biodiesel was removed exactly with that ratio. The temperature was selected in order to minimize process costs by avoiding heating of the system. The short residence time was chosen to ensure fast screening of selected DESs and based on the previous research [8] where glycerol was removed from biodiesel in 0.22 min. In addition, a longer residence time was not chosen at that point simply to avoid complete glycerol extraction because in that way it would not be possible to choose the best DES. The obtained results are presented in Figure 2A,B.

As can be seen from these preliminary results, all of the selected eutectic solvents removed glycerol from biodiesel, with the lowest extraction efficiency obtained with ChCl:PG:ZnCl_2_, while the highest extraction efficiency was observed when ChCl:Gly (1:3), ChlCl:EG (1:3) and B:PG (1:3.5) were used. The presented results are in correspondence with the results of our previous research [8,48] where ChCl:Gly and ChlCl:EG were successfully used for glycerol removal.

In addition, all prepared DESs have a very low freezing point [11]. Due to the polarity of DESs, the existence of hydroxyl groups in both DES (solvent) and glycerol (solute), and the solvation power for glycerol in biodiesel, DESs have a high affinity for attracting glycerol through hydrogen bonding and dipole-dipole attraction. All this leads to improved extractability of the solvent [11].

When the obtained results (Figure 2B) for mass fractions of glycerol are observed, it can be seen that the glycerol amounts in biodiesel samples do not correspond to the values prescribed by the ASTM D6751 and EN 14214 standards. Due to that, further optimization of the extraction process was necessary. Among several DES candidates that proved effective in the removal of glycerol, ChCl:EG was chosen for further extraction optimization experiments due to its lowest viscosity, which assured the simplest possible operation of a microextractor. As the ethylene glycol mole fraction increases, the viscosity of the DESs gradually decreases, which is related to the intrinsically low viscosity of ethylene glycol.

### 3.4. Influence of Deep Eutectic Solvents Properties on Glycerol Extraction

The extraction efficiency is related to the properties of the DES used and it was defined which physicochemical properties of DES have the most influence on the extraction efficiency. To get insight into relationship between DESs properties and extraction efficiency, the regression analysis was used. Firstly, the relationship between DESs molecular descriptors and extraction efficiency was evaluated. Molecular descriptors can be defined as mathematical representations of molecules properties that are generated by algorithms [49] and there is still a big challenge in development and selecting of the specific one for each particular application [50]. According to Abranches et al. [26], *σ*-profile was presented as a potentially useful choice for a universal molecular descriptor. In this work, firstly, it was assumed that the extraction efficiency can be expressed as a function of the *σ*-profile of the mixture, expressed by a set of *S^i^*_mix_ descriptors. To select the appropriate *S^i^*_mix_ descriptors, mostly contributing the extraction efficiency, Spearman correlations between *S^i^*_mix_ descriptors and extraction efficiency were analyzed. As presented in Table 3, significant correlations were noticed between *S*^1^_mix_, *S*^3^_mix_, *S*^4^_mix_, *S*^5^_mix_, *S*^6^_mix_ and extraction efficiency and therefore those *S^i^*_mix_ descriptors were used for further modeling. The similar approach to model input variables selection was previously described by Benguerba et al. [27] and Panić et al. [30]. By reducing the number of model input variables by excluding the non-significant ones, a low dispersion between observed DES viscosity data and multiple linear region model data was achieved.

**Figure 3:**
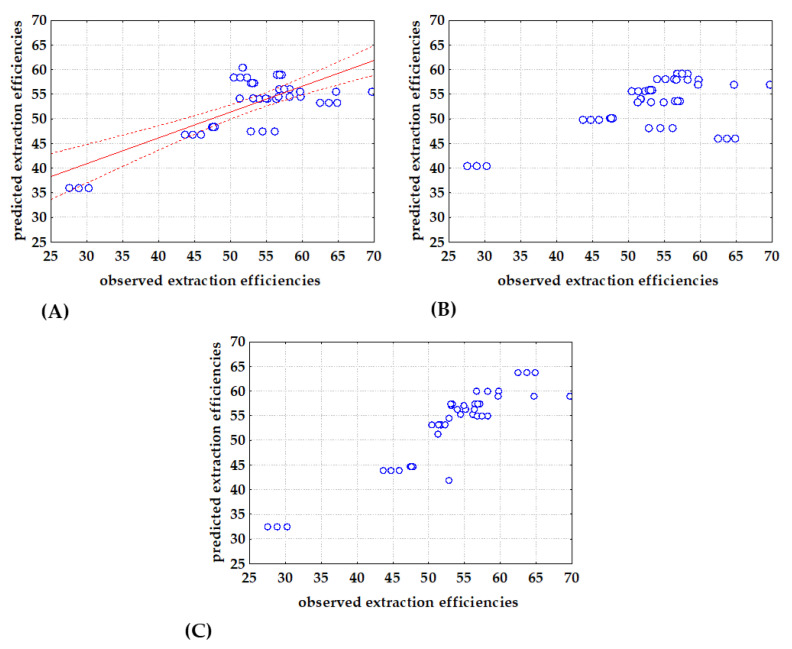
Comparison between observed and (**A**) MLR, (**B**) NLR and (**C**) PLR predicted extraction efficiencies based on DESs *S^i^*_mix_ descriptors (−regression line, ⋅⋅⋅⋅ 95 % confidence interval).

The success of MLR, NLR and PLR models to describe the extraction efficiency of DESs was evaluated using *R*^2^, *R*^2^_adj_ and RMSE. As stated by Sanquetta et al., [51] criteria for model selection must incorporate goodness-of-fit allowing that several models examined can be simultaneously compared. Estimated model coefficients are given in Table 4 and comparisons between observed and model predicted data are shown in Figure 3. It can be noticed that the best agreement between observed and model predicted data was obtained with the PLR model, while the biggest dispersion between observed and model predicted data was found with the MLR model. It is also important to emphasize that, for all the three analyzed models, all estimated coefficients were significant, indicating that the most important descriptors were used as the model’s input variables. It can also be noticed that estimated coefficients of the MLR, NLR and PLR models have the same trend. Coefficients *b*_1_, *b*_3_ and *b*_5_ related with *S*^1^_mix_, *S*^4^_mix_ and *S*^6^_mix_ had negative values, while coefficients related with *S*^3^_mix_, and *S*^5^_mix_ had positive values. As described by Zhang and Li [52], in the piecewise-regression analysis (also known as segmented regression) a dataset is split at a defined break point, and regression parameters (intercept and slopes) are calculated separately for data before and after the breakpoint. Piecewise linear regression is applicable if the data exhibit different linear trends over different domains [53]; thus, the regression can be made more accurate, as it is the case in this work (Figure 3C). By analyzing Figure 3A,B, it can be noticed that data behave differently below and above the 50% efficiency threshold, which was confirmed by the estimated break point at 52.9 ± 5.1 PLR models were also previously shown to be more efficient that MLR models for the prediction of the DESs pH values based on the *σ*-descriptors [30].

**Figure 4:**
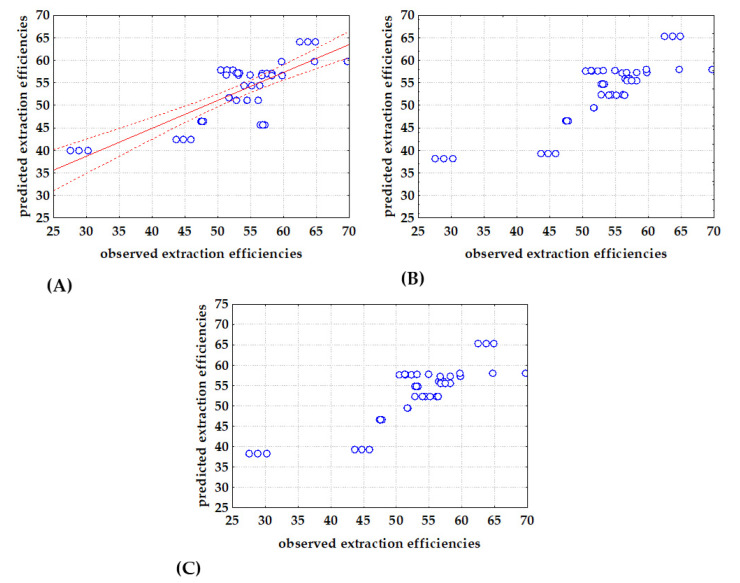
Comparison between observed and (**A**) MLR, (**B**) NLR and (**C**) PLR predicted extraction efficiencies based on DESs physical properties (−regression line, ⋅⋅⋅⋅ 95 % confidence interval).

Taking into account the statement of Le Man et al. [54] that a regression model can be considered applicable if *R*^2^ is greater than 0.75, the models based on *σ*-descriptors should be further improved. Therefore, a second approach was evaluated, where glycerol extraction efficiency (*E*) was modeled as the function of DESs physical properties including: density (*ρ*), dynamic viscosity (*η*), specific conductance (*σ*), refractive index (*n*_D_), thermal diffusivity (*a*), dynamic viscosity (*λ*), and specific heat capacity (*c*_p_). Estimated model coefficients are given in Table 5 and comparisons between observed and model predicted data are shown in Figure 4. As for the models based on *σ*-descriptors, the best agreement between experimental data and model predicted data (the highest *R*^2^ > 0.9 and the lowest RMSE) was obtained for the PLR model (Figure 4C). Furthermore, estimated regression coefficients showed that density, electrical conductivity, refractive index, thermal conductivity and specific heat capacity had negative effects on the extraction efficiency, while dynamic viscosity and thermal diffusivity exhibited positive effects. Also, in the cases of MLR and PLR, all coefficients were significant, while in the case of NLR model, coefficients *b*_3_ and *b*_4_ describing the effects of specific conductance and refractive index, respectively, were non-significant. Moreover, ANOVA analysis showed that all developed models, including those based on *σ*-descriptors and those based on DESs physical properties, were significant with *p* < 0.05 and F-values higher that F-critical = 2.29 (Table 5).

### 3.5. Extraction Optimization

After selecting the best DES for glycerol extraction, the next step was the optimization of the extraction conditions since at this point mass fraction of glycerol was still above the values prescribed by the ASTM D6751 and EN 14214 standards. The extraction optimization was performed in a continuously operated PTFE microextractor with two inlets and a T-junction. The effects of the (i) extraction temperature *X*_1_ (*T* = 25, 40, 55 °C), (ii) residence time *X*_2_ (*τ* = 0.05, 0.5, 0.95 min) and (iii) volume ratio of biodiesel:DES X_3_ (9:1, 1:1, 1:9 *v*/*v*) were analyzed. The results obtained according to Box-Benkhen experimental design are presented in Table 6. Extraction efficiencies achieved were in the range from *E* = 26.3 ± 3.9 % to *E* = 57.2 ± 0.5%. The lowest extraction efficiency was obtained for *T* = 25 °C, *τ* = 0.50 min and biodiesel:DES ratio of 90:10, while the highest extraction efficiency was obtained for *T* = 40 °C, *τ* = 0.50 min and biodiesel:DES volume ratio of 1:9.

The obtained results were as expected. Several factors influence the extraction processes while the extraction temperature, extraction time, characteristics of solid particles (size, shape, and condition), and type of solvent are the most important ones [8].

Moreover, the DES/biodiesel molar ratio has a positive effect on the overall glycerol removal efficiency. This means that if more DES is available, more glycerol will be removed.

**Figure 5:**
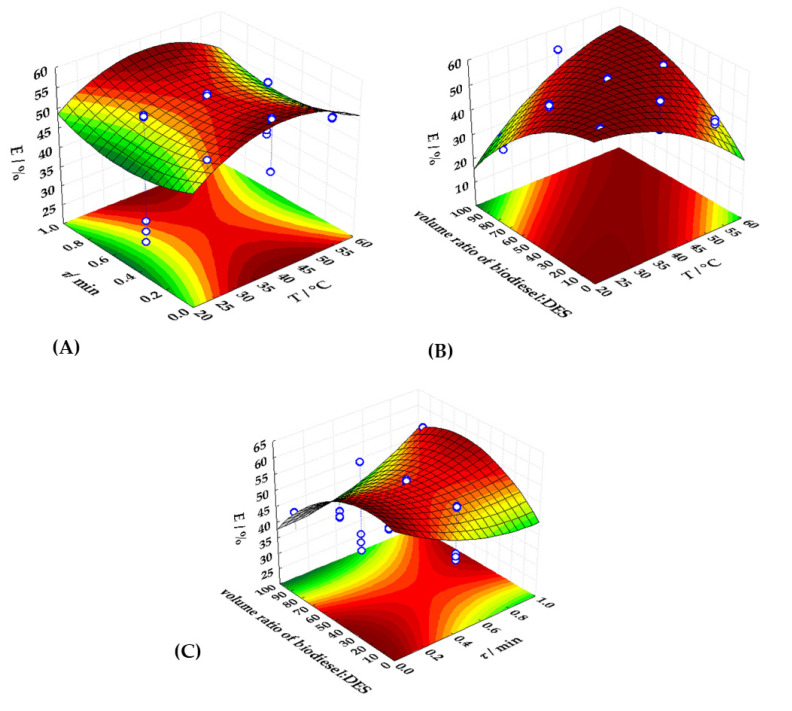
Three dimensional response surface plot for the interaction between (**A**) extraction temperature and residence time, (**B**) extraction temperate and biodiesel:DES volume ratio and (**C**) residence time and biodiesel:DES volume ratio.

To identify variables with a significant effect on the response variable, a second-order polynomial model was used to describe the experimental data. The regression coefficients of the developed models are given in Table 7. The results showed the significant influence of residence time and biodiesel:DES volume ratio in linear and quadratic coefficients and the significant effect of temperature in the quadratic coefficient of RSM model for extraction efficiency. By analyzing results presented in Table 7, it can be noticed that the residence time and biodiesel:DES volume ratio have a negative effect on the extraction efficiency, while temperature has a positive effect. Figure 5 depicts 3D response surfaces, which illustrate the interaction effects of the independent factors on the extraction efficiency. The graphs were created by correlating the response of two independent variables (the third was kept constant). It can be noticed that the extraction efficiency increases with temperature until optimum temperature is reached. Moreover, according to the *R*^2^ value and RMSE of the proposed RSM model, it could be assumed that the RSM model accurately describes the experimental data. ANOVA revealed that the proposed model was significant (*p* < 0.05) and that the F-value for the model was higher than the F-critical (2.8).

**Figure 6:**
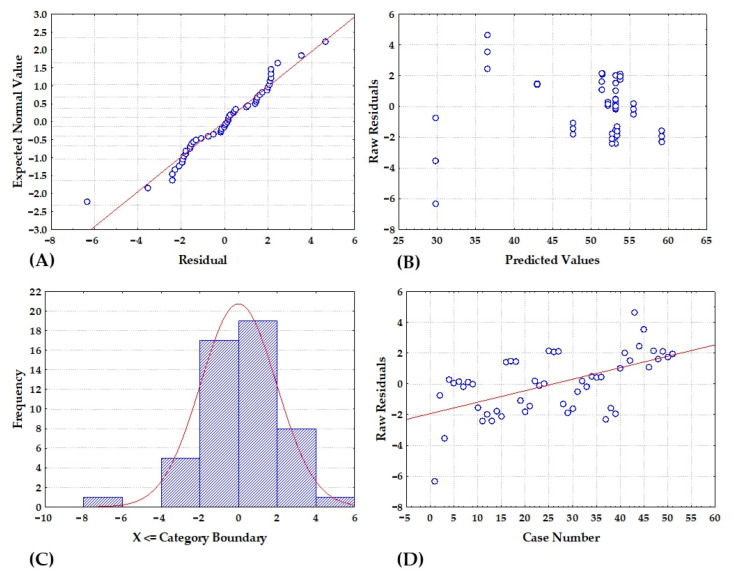
RSM model residual analysis (**A**) normality plot, (**B**) dependence between model predicted values and residuals, (**C**) histogram of residuals and (**D**) dependence between experiment number and residual value.

Given that a high *R*^2^ value does not ensure that the model would fit the data well, residual analysis was also carried out. The results of the residual analysis are shown in Figure 6. Residuals were distributed approximately around the line (Figure 6A), and histograms depicting residual classification (Figure 6C) exhibited a distinctive bell shape, confirming the assumption of normality. Furthermore, by examining the plots of residuals vs. estimated values (Figure 6B), it is clear that the residuals were randomly distributed, indicating high agreement between the model and the experimental data. The residual analysis further revealed that the sequence of the experimental runs had no effect on the results, since the residuals distributed themselves around zero (Figure 6D). The acquired results suggest that the proposed response surface model was reliable for the examined range of input variables.

The optimal conditions were those that resulted in the highest extraction efficiency. According to RSM model, the optimal extraction conditions were *T* = 55 °C, *τ* = 0.95 min and biodiesel:DES volume ratio of 1:9. The optimal extraction conditions were estimated based on the desirability profiles obtained from the RSM predictions. The desirability scale ranged from 0 (undesirable) to 1 (very desirable). The estimated optimal conditions represent the local maximum for the selected range of input variables.

**Figure 7:**
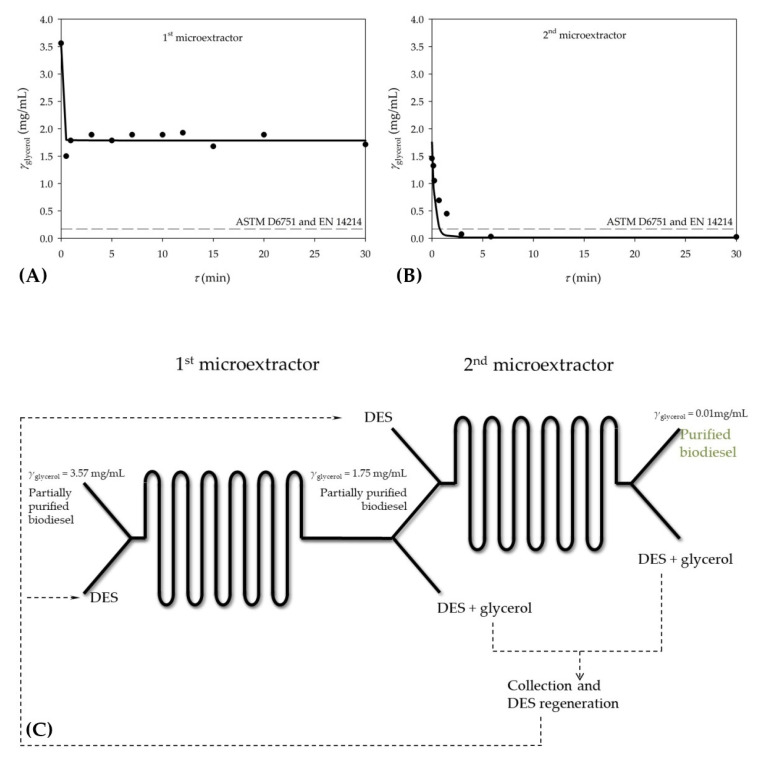
Influence of the residence time on the extraction of glycerol from biodiesel using DES as a solvent (**A**) glycerol concentration in 1st microextractor, (**B**) glycerol concentration in a 2nd microextractor and (**C**) proposed system composed of two microextractor connected into series for extraction of glycerol from biodiesel [▬ mathematical model, ● experimental results (DES phase)].

After optimizing the process conditions, the biodiesel purification process was carried out in a microextractor with the selected DES under optimal conditions to validate the mathematical model of the process. The obtained experimental results were compared with the simulation of the mathematical model. According to the mathematical model, it was predicted that 60.6% of glycerol would be removed from the biodiesel under optimal conditions. The experimental results showed that 53 ± 5.2% (*w* = 0.2 ± 0.1 *w*/*w*) of glycerol was removed during extraction under optimal conditions. Although there was a good agreement between the model and the experimental results, the results were not satisfactory since the mass fraction of glycerol was still higher than the values prescribed by ASTM D6751 and EN 14214 standards. Considering that the results under optimal conditions did not meet the standard requirements, the process of purification of biodiesel with eutectic solvent was carried out in a microextractor with a smaller channel diameter to increase the rate of glycerol transfer as a result of a shorter diffusion path, which should lead to higher extraction efficiency. This assumption was based on our previous work [8], where glycerol extraction was performed in three different microextractor channel sizes (250, 350 and 500 µm). Therein it was demonstrated that the channel diameter had a significant effect on the extraction efficiency. When the extraction was performed in the narrowest channel, the maximum extraction efficiency was obtained in 13.61 s and in the widest channel it took 55.03 s to obtain the same efficiency. In this study, up to this point, only the microextractor with the channel diameter of 1000 µm was used; however, new experiments were performed with the 500 µm diameter microchannel. When extraction was performed in a narrower channel, only a 4 percent point increase in extraction efficiency was observed. In addition to analyzing the influence of the diameter of the microextractor channel on the extraction efficiency, the influence of the residence time on the success of glycerol extraction was also investigated. It was found that the residence time in the studied range (0.5–30 min) had no significant influence on the extraction process in the microextractor, i.e., the achieved extraction efficiencies did not differ significantly among the residence times. At this point, it became clear that despite the change in extraction conditions, a higher efficiency of the process would not be achieved. However, comparing the obtained results with the previous studies [8,26,46], in which successful glycerol purification was achieved, it was observed that initial glycerol concentration in this study was significantly higher than in the previous ones. In this study, the glycerol concentration at the inlet of microextractor was 3.57 ± 0.78 mg/mL, while in the previous studies it was 0.8 mg/mL or 1.5 mg/mL. One has to understand that the compositions of the biodiesel and DES phase will approach equilibrium in the cases of sufficiently long microchannels or sufficiently low flowrates. Thus, molar fractions cannot exceed the equilibrium ones and, the higher is the initial molar fraction of glycerol on biodiesel, the higher are the equilibrium molar fractions of glycerol in both phases. But such high molar fractions of glycerol in biodiesel might exceed the standard requirements. It might be necessary to perform the extraction in more than a single stage. To estimate how many microextractors are required for the sufficient removal of glycerol, a mathematical model was used that has been described and validated elsewhere [8]. According to the mathematical model, two microextractors should be connected in series to remove the glycerol so that biodiesel can meet ASTM D6751 and EN 14214 standards (Figure 7A,B). To validate this, the outlet stream from the 1st microextractor was collected and biodiesel was separated from DES. Collected biodiesel, now containing 1.75 ± 0.21 mg/mL glycerol was introduced into the 2nd microextractor together with the fresh DES. As it can be seen from Figure 7B, predictions of mathematical model were validated with the experimental results and biodiesel of sufficient purity was obtained at the exit of microextractor.

Based on the obtained results, an integrated system (Figure 7C) was proposed as a possible solution for continuous glycerol extraction. In that system, partially purified biodiesel would enter the microextractor as one process stream and DES as the other one. As reported in our previous work [8], a stable and parallel flow is formed in this system, with the interface located exactly in the middle of the microextractor channel. This allows for complete phase separation at the outlet of the microextractor. Biodiesel that now contains 50% less glycerol than partially purified biodiesel at the inlet of the 1st microseparator is fed into the 2nd microextractor along with fresh DES, resulting with purified biodiesel at the exit. All DES waste streams could be collected, regenerated and returned to the process, making the proposed system more sustainable.

It is also noted that the extraction of glycerol is very fast in both microextractors. As it can be seen, the desirable extraction efficiency was reached after only 0.5 min in the 1st microextractor, as well as in the 2nd microextractor. This indicates that if the flow rate is chosen correctly and biodiesel can be completely purified in about one minute with microextractors connected in series, making this process significantly faster than other biodiesel purification processes.

## 4. Conclusions

In this work, optimization of the biodiesel purification process using eutectic solvents in a microextractor was carried out to obtain biodiesel suitable for use in internal combustion engines. Mathematical models were used to determine which physical and chemical properties of DES and to what extent affect the efficiency of extraction of glycerol from biodiesel. It was found that the physical and chemical properties of DES have an impact on glycerol separation. It was found that increasing density, electrical conductivity, refractive index, thermal conductivity and specific heat capacity negatively impact the extraction efficiency, while increasing dynamic viscosity and thermal diffusivity affects it positively. Among the 12 DES candidates, which allowed for the effective removal of glycerol, the highest extraction efficiency was observed when ChCl:Gly (1:3), ChlCl:EG (1:3) and B:PG (1:3.5) DESs were used. DES ChCl:EG (1:3) was chosen for further extraction optimization experiments due to its high extraction efficiency and the lowest viscosity compared to three mentioned DESs, which makes the microextractor operation rather simple. To determine the optimal process conditions (temperature, biodiesel:DES volume ratio, residence time), the three-level-three-factor Box-Behnken experimental design was used. The highest extraction efficiency was obtained at *T* = 40 °C, *τ* = 0.50 min, and biodiesel:DES volume ratio of 1:9. After optimizing the conditions, the biodiesel purification process was carried out in a microextractor with the selected DES under optimal conditions and only 52.99 ± 5.23% (*w* = 0.19 ± 0.12 *w*/*w*) of glycerol was removed. In addition, different microchannel diameters and different residence times were tested, which unfortunately also led to unsatisfactory results. As a possible solution, an integrated system, consisting of two microextractors connected in a series, was proposed for glycerol removal. The results obtained indicated that this new approach could be a good solution for glycerol removal, since the biodiesel was almost completely purified in about one minute, making this process much faster than other biodiesel purification methods.

## Figures and Tables

**Table 1:** Prepared DESs.

Deep Eutectic Solvent	Abbreviation	Hydrogen Bond Acceptor	Hydrogen Bond Donor	Acceptor:Donor Molar Ratio
Choline chloride:glycerol	ChCl:Gly	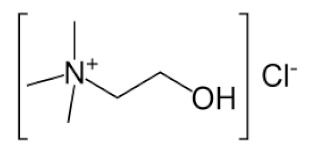	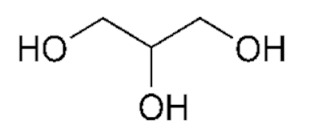	1:2 1:3
Choline chloride:ethylene glycol	ChCl:EG	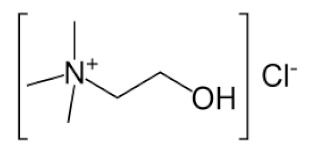	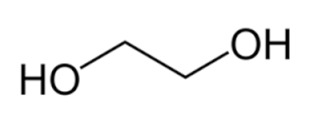	1:2 1:3
Betaine:glycerol	B:Gly	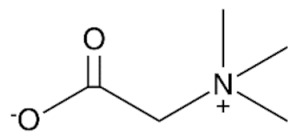	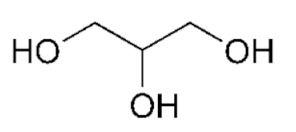	1:3 1:4
Betaine:ethylene glycol	B:EG	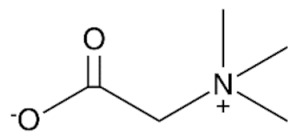	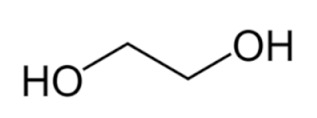	1:3
Betaine:propylene glycol	B:PG	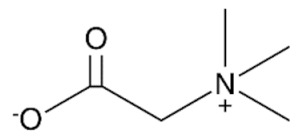	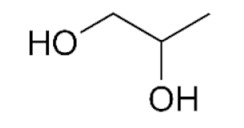	1:3.5
Choline chloride:propylene glycol	ChCl:PG	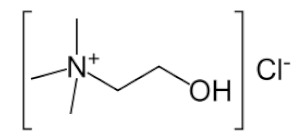	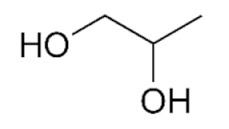	1:3 1:4
Choline chloride:propylene glycol:zinc chloride	ChCl:PG:ZnCl_2_	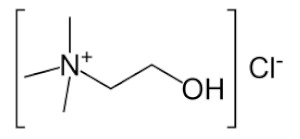	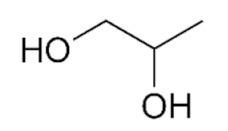	1:4:0.02
Betaine:glycerol:zinc chloride	B:Gly:ZnCl_2_	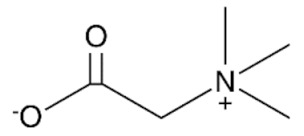	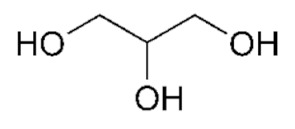	1:4:0.02

**Table 2:** Properties of selected DESs at 25 °C.

DES	*ρ*, g/mL	*η*, Pa s	*σ*, mS/cm	*n_D_*	*a*, mm^2^/s	*λ*, W/(m K)	*c_p_*, J/(g K)	Ref.
ChCl:Gly (1:2)	1.188 ± 0.002	0.369 ± 0.053	1.130 ± 0.010	1.448 ± 0.000	0.097 ± 0.011	0.232 ± 0.006	2.010 ± 0.193	[22]
ChCl:Gly (1:3)	1.204 ± 0.001	0.316 ± 0.011	1.122 ± 0.008	1.448 ± 0.000	0.097 ± 0.009	0.241 ± 0.005	2.057 ± 0.140	[22]
ChCl:EG (1:2)	1.115 ± 0.002	0.042 ± 0.000	8.610 ± 0.005	1.448 ± 0.000	0.167 ± 0.002	0.227 ± 0.002	1.205 ± 0.003	[23]
ChCl:EG (1:3)	1.113 ± 0.000	0.028 ± 0.000	9.410 ± 0.010	1.448 ± 0.000	0.195 ± 0.020	0.231 ± 0.008	1.055 ± 0.100	[23]
B:Gly (1:3)	1.223 ± 0.000	1.103 ± 0.012	0.001 ± 0.000	1.478 ± 0.000	0.151 ± 0.011	0.270 ± 0.004	1.455 ± 0.091	[24]
B:EG (1:3)	1.131± 0.000	0.062 ± 0.002	0.006 ± 0.001	1.456 ± 0.000	0.189 ± 0.005	0.231 ± 0.002	1.071 ± 0.018	[24]
B:PG (1:3.5)	1.074 ± 0.000	0.139± 0.003	0.000 ± 0.000	1.452 ± 0.000	0.116 ± 0.003	0.206 ± 0.093	1.642 ± 0.048	[24]
ChCl:PG (1:3)	1.078 ± 0.003	0.066 ± 0.004	3.380 ± 0.009	1.458 ± 0.000	0.145 ± 0.023	0.208 ± 0.013	1.347 ± 0.147	[23]
ChCl:PG (1:4)	1.075 ± 0.134	0.049 ± 0.000	3.093 ± 0.033	1.455 ± 0.000	0.217 ± 0.021	0.213 ± 0.007	0.915 ± 0.063	
ChCl:PG:ZnCl_2_ (1:4:0.02)	1.079 ± 0.000	0.054 ± 0.002	2.290 ± 0.073	1.455± 0.000	0.219 ± 0.001	0.211 ± 0.003	0.896 ± 0.034	
B:Gly (1:4)	1.232 ± 0.006	2.431 ± 0.006	2.920 ± 0.021	1.456 ± 0.000	0.138 ± 0.017	0.278 ± 0.006	1.653 ± 0.173	
B:Gly:ZnCl_2_ (1:4:0.02)	1.233 ± 0.000	1.406 ± 0.396	2.770 ± 0.029	1.456 ± 0.000	0.094 ± 0.016	0.257 ± 0.100	2.257 ± 0.355	

**Table 3:** Correlation matrix for *S^i^*_mix_ descriptors and extraction efficiencies (correlations significant for *p* < 0.05 are marked bold).

	*S* ^1^_mix_	*S* ^2^_mix_	*S* ^3^_mix_	*S* ^4^_mix_	*S* ^5^_mix_	*S* ^6^_mix_	*S* ^7^_mix_	*S* ^8^_mix_	*S* ^9^_mix_	*S* ^10^_mix_	*E*
*S* ^1^_mix_	1.000	0.260	0.038	**−0.439**	0.287	**0.357**	**0.341**	0.095	−0.299	0.085	**−0.489**
*S* ^2^_mix_	0.260	1.000	**0.848**	−0.050	−0.114	−0.012	**0.852**	**0.843**	0.131	0.170	0.247
*S* ^3^_mix_	0.038	**0.848**	1.000	**0.454**	**−0.342**	−0.258	**0.841**	**0.984**	**0.636**	−0.219	**0.312**
*S* ^4^_mix_	**−0.439**	−0.050	**0.454**	1.000	**−0.647**	**−0.689**	0.011	**0.373**	**0.903**	**−0.418**	**0.348**
*S* ^5^_mix_	0.287	−0.114	**−0.342**	**−0.647**	1.000	**0.984**	0.077	−0.242	**−0.419**	0.005	**−0.438**
*S* ^6^_mix_	**0.357**	−0.012	−0.258	**−0.689**	**0.984**	1.000	0.207	−0.137	**−0.409**	−0.062	**−0.470**
*S* ^7^_mix_	**0.341**	**0.852**	**0.841**	0.011	0.077	0.207	1.000	**0.913**	**0.356**	**−0.323**	−0.001
*S* ^8^_mix_	0.095	**0.843**	**0.984**	**0.373**	−0.242	−0.137	**0.913**	1.000	**0.620**	**−0.330**	0.218
*S* ^9^_mix_	−0.299	0.131	**0.636**	**0.903**	**−0.419**	**−0.409**	**0.356**	**0.620**	1.000	**−0.681**	0.201
*S* ^10^_mix_	0.085	0.170	−0.219	**−0.418**	0.005	−0.062	**−0.323**	**−0.330**	**−0.681**	1.000	0.248
*E*	**−0.489**	0.247	**0.312**	**0.348**	**−0.438**	**−0.470**	−0.001	0.218	0.201	0.248	1.000

**Table 4:** MLR, NLR and PLR coefficients for prediction of extraction efficiencies based on DESs *S^i^*_mix_ descriptors (significant coefficients for *p* < 0.05 are marked bold).

	MLR	NLR	PLR
Break point			**52.9 ± 5.1**
*b*_0_	−6.1 ± 4.1	3.9 ± 1.7	**−9.0 ± 1.1** **40.7 ± 7.9**
*b*_1_ (*S*^1^_mix_)	**−184.1 ± 59.2**	**−0.08** **± 0.03**	**−201.2 ± 10.3** **−103.1 ± 11.8**
*b*_2_ (*S*^3^_mix_)	**3.6 ± 1**	**1** **± 0.4**	**4.6 ± 0.6** **0.3 ± 1 × 10^−2^**
*b*_3_ (*S*^4^_mix_)	**−1.8 ± 0.6**	**−0.5** **± 0.1**	**−1.1 ± 0.1** **−0.6 ± 1 × 10^−2^**
*b*_4_ (*S*^5^_mix_)	**5.5 ± 2.2**	**0.4 ± 0.1**	**2.3 ± 0.4** **0.8 ± 2 × 10^−2^**
*b*_5_ (*S*^6^_mix_)	**−9.2 ± 3.2**	**−0.03 ± 0.01**	**−3.0 ± 0.2** **−0.4 ± 4 × 10^−3^**
*R* ^2^	0.52	0.67	0.73
*R* ^2^_adj_	0.46	0.52	0.68
RMSE	6.39	5.36	4.47
F−value	F (5,36) = 7.93	F (5,36) = 7.93	F (5,36) = 7.93
*p*−value	*p* < 0.001	*p* < 0.001	*p* < 0.001

**Table 5:** MLR, NLR and PLR coefficients for prediction of extraction efficiencies based on DESs physical properties (significant coefficients for p < 0.05 are marked bold).

	MLR	NLR	PLR
Break point			**52.9 ± 5.1**
*b*_0_	**2489 ± 561.4**	1.4 ± 0.9	**1310.6 ± 157.1** **−4315.7 ± 237.2**
*b*_1_ (*ρ*)	**−292.3 ± 83.8**	**−17.9 ± 4.3**	**−2604.4 ± 188.9** **−367.9 ± 22.5**
*b*_2_ (*η*)	**2 × 10^−2^** **±** **1 × 10^−3^**	**−0.1 ± 3** **×** **10^−2^**	**2 × 10^−2^ ± 1 × 10^−3^** **−46.9 ± 21.1**
*b*_3_ (σ)	**−0.7 ± 0.5**	−1 × 10^−2^ ± 3 × 10^−3^	**5.2 ± 2.1** **1.5 ± 0.6**
*b*_4_ (*n*_D_)	**−1573.7 ± 371.3**	20.7 ± 1.7	**−7991.6 ± 255.6** **3084.7 ± 154.1**
*b*_5_ (*a*)	**−220.5 ± 97.3**	**−13.9 ± 4.1**	**694.9 ± 115.7** **−317.5 ± 59.7**
*b*_6_ (*λ*)	**738.1 ± 18.4**	**16.6 ± 4.22**	**4789.4 ± 321.5** **1455.2 ± 118.7**
*b*_7_ (*c_p_*)	**−3.2 ± 1.2**	**−13.5 ± 3.9**	**217.2 ± 15.8** **24.2 ± 3.8**
*R* ^2^	0.62	0.73	0.97
*R* ^2^_adj_	0.51	0.68	0.97
RMSE	5.89	4.47	1.45
F−value	F (7,34) = 7.9	F (7,34) = 7.9	F (7,34) = 7.9
*p*−value	*p* < 0.001	*p* < 0.001	*p* < 0.001

**Table 6:** Optimization of extraction by Box-Behnken experimental design (observed response factor levels are shown in parentheses).

Exp.	*T*/°C	*τ*/min	Volume Ratio of Biodiesel:DES	*E*/%
1	25.00 (−1)	0.50 (0)	9:1 (1)	26.9 ± 3.9
2	55.00 (1)	0.50 (0)	9:1 (1)	52.3 ± 0.2
3	40.00 (0)	0.50 (0)	1:1 (0)	53.1 ± 0.2
4	40.00 (0)	0.50 (0)	1:1 (0)	51.2 ± 0.6
5	55.00 (1)	0.95 (1)	1:1 (0)	50.6 ± 0.4
6	40.00 (0)	0.05 (−1)	9:1 (1)	44.4 ± 0.1
7	40.00 (0)	0.95 (1)	1:9 (−1)	46.2 ± 0.5
8	40.00 (0)	0.50 (0)	1:1 (0)	53.2 ± 0.2
9	25.00 (−1)	0.05 (−1)	1:1 (0)	53.5 ± 0.1
10	55.00 (1)	0.05 (−1)	1:1 (0)	51.7 ± 0.4
11	25.00 (−1)	0.50 (0)	1:9 (−1)	55.3 ± 0.5
12	40.00 (0)	0.50 (0)	1:1 (0)	53.6 ± 0.1
13	40.00 (0)	0.05 (−1)	1:9 (−1)	57.2 ± 0.5
14	40.00 (0)	0.50 (0)	1:1 (0)	54.6 ± 0.7
15	55.00 (1)	0.50 (0)	1:9 (−1)	40.1 ± 1.6
16	25.00 (−1)	0.95 (1)	1:1 (0)	52.9 ± 0.6
17	40.00 (0)	0.95 (1)	9:1 (1)	55.7 ± 0.3

**Table 7:** RSM model for description of glycerol extraction efficiency.

Coefficient	Regression Coefficient ± St. Error	*p*-Value
*β*_0_	61.9 ± 5.2	<0.001
*β*_1_ (*T*)	0.7 ± 0.2	0.005
*β*_2_ (*τ*)	−2 × 10^−2^ ± 3 × 10^−3^	<0.001
*β*_3_ (*v*/*v*)	−30.9 ± 5.2	<0.001
*β*_4_ (*T^2^*)	15.9 ± 3.0	<0.001
*β*_5_ (*τ*)	−0.6 ± 6 × 10^−2^	<0.001
*β*_6_ (*v*/*v*)	−3 × 10^−3^ ± 1 × 10^−3^	<0.001
*β*_7_ (*T* × *τ*)	−2 × 10^−2^ ± 1 × 10^−2^	0.823
*β*_8_ (*T* × *v*/*v*)	2 × 10^−2^ ± 1 × 10^−3^	<0.001
*β*_9_ (*τ* × *v*/*v*)	0.3 ± 4 × 10^−2^	<0.001
RSM model	*R* ^2^	0.93
	*R* ^2^_adj_	0.91
	RMSE	1.96
	F-value	10.12
	*p*-value	<0.001

## Data Availability

Not applicable.
